# Effects of Raloxifene on Bone in Patients With Type 2 Diabetes

**DOI:** 10.5812/ijem.6558

**Published:** 2012-09-30

**Authors:** Itsuo Gorai

**Affiliations:** 1 Department of Obstetrics and Gynecology, Hori Hospital, Futatsubashi-Cho, Seya-ku, Yokohama, Japan

**Keywords:** Type 2 Diabetes, Raloxifene, Hemodialysis

Dear Editor,

Raloxifene, one of selective estrogen receptor modulators, has been widely prescribed to relatively early postmenopausal women with osteoporosis and osteopenia since postmenopausal women on hormone therapy (HT) stopped the medication, and the number of postmenopausal women starting HT decreased, mainly because of the increased risks of breast cancer and cardiovascular diseases after the Women’s Health Initiative (WHI) report (1).

Saito et al. showed that reduction in BMD in postmenopausal dialysis women both with and without type 2 diabetes was suppressed by raloxifene therapy, indicating raloxifene treatment is useful and could be a possibility for women with type 2 diabetes (2). Before we reach the conclusion, I am afraid that there might be some problems that have to be made clear although the trial was well organized. First, the number of subjects is very small (100 or fewer). Second, the subjects are rather early postmenopausal women (range: 50-87 years). The criteria of postmenopausal status were not defined. Third, do 19 diabetic subjects on diet therapy alone really need hemodialysis? Fourth, the values of HbA1c are not shown in non-diabetic group. Fifth, the speed of sound (SOS) does not measure BMD although the SOS has been shown to be significantly correlated with lumbar BMD. Finally, the changes in serum Ca, P, intact PTH (i-PTH) and BAP have not been evaluated in the text.

We have recently shown that raloxifene alone could induce a significant increase in circulating i-PTH, which might diminish the beneficial effect of raloxifene on BMD, and that the addition of alfacalcidol to raloxifene demonstrated a greater bone sparing effect by normalizing serum i-PTH level than raloxifene alone therapy in postmenopausal Japanese women with osteoporosis or osteopenia (3). In our study i-PTH significantly increased and corrected serum calcium significantly decreased at 6 month and thereafter in subjects on raloxifene alone therapy, which might be caused by inhibition of bone resorption. Our result is not in agreement with the reports that raloxifene lowered serum PTH in postmenopausal women with osteoporosis (4) and that raloxifene did not affect PTH levels in those with primary hyperparathyroidism (5) although serum calcium decreased in both studies. However the authors did not mention whether raloxifene has any effects on i-PTH secretion and serum Ca, i-PTH in diabetic patients was lower than in non-diabetic patients after one year. As shown in tables 1 and 2 (2), i-PTH levels decreased one year after raloxifene therapy (54.7 ng/L) in diabetic subjects compared to control (71.9 ng/L) or baseline (78.2 ng/L), suggesting that raloxifene might have some influences on i-PTH secretion, possibly suppressing i-PTH secretion where hemodialysis patients have secondary hyperparathyroidism. In contrast, corrected serum Ca levels did not change remarkably after raloxifene therapy (2.27 mmol/L) vs. control (2.13 mmol/L) or baseline (2.22 mmol/L). The discrepancy between this study and our study could be explained by the difference in vitamin D status of the subjects; in this study vitamin D is co-administered (vitamin D replete) whereas in our study it is not (vitamin D insufficiency or deficiency), suggesting that raloxifene might exert its inhibitory effect on PTH secretion only when vitamin D status is replete or sufficient.

In any way this study shows that raloxifene therapy could be useful in postmenopausal dialysis patients both with and without type 2 diabetes.
